# Dose–Response Relationship Between Triglyceride Glucose‐Waist Height Ratio Index and Cardiometabolic Multimorbidity Among Chinese Middle‐Aged and Older People

**DOI:** 10.1002/jcla.70389

**Published:** 2026-10-30

**Authors:** Lingling Li, Zongliang Yu, Yixuan Feng, Changxin Sun, Lanqing Hu, Xinyi Liu, Liang Li, Min Wu, Longtao Liu

**Affiliations:** ^1^ Department of Cardiology Guang'anmen Hospital, China Academy of Chinese Medical Sciences Beijing China; ^2^ National Clinical Research Center for Chinese Medicine Cardiology Xiyuan Hospital, China Academy of Chinese Medical Science Beijing China; ^3^ Graduate School Beijing University of Chinese Medicine Beijing China; ^4^ Geriatrics Department Zhongshan Hospital of Traditional Chinese Medicine Zhongshan China

**Keywords:** cardiometabolic multimorbidity, CHARLS, dose–response relationship, risk prediction, the triglyceride glucose‐waist height ratio (TyG‐WHtR) index

## Abstract

**Background:**

Cardiometabolic multimorbidity (CMM) is a growing public health challenge. The triglyceride glucose‐waist height ratio (TyG‐WHtR) is a novel index linked to metabolic disorders. This study explores the dose–response relationship between TyG‐WHtR and CMM.

**Methods:**

Data from the China Health and Retirement Longitudinal Study (CHARLS), including the 2011 baseline survey and the 2018 follow‐up survey with an average follow‐up duration of 7.0 years, were analyzed. Multivariate logistic regression and restricted cubic spline models assessed the TyG‐WHtR–CMM relationship, adjusting for covariates.

**Results:**

The study enrolled 6674 individuals, and 1140 patients with cardiometabolic multimorbidity and 5534 patients with non‐cardiometabolic multimorbidity were documented over an average follow‐up duration of 7.0 years. The fourth TyG‐WHtR quartile group (Q4) had the highest CMM risk, with an odds ratio of 1.23 and a 95% confidence interval of 1.20–1.26. The restricted cubic spline regression model showed a nonlinear (*p* < 0.001) association between TyG‐WHtR and CMM. Subgroup analyses showed that the positive association between TyG‐WHtR and CMM was evident among females and ex‐smokers. A notable interaction was observed between TyG‐WHtR and variables like age and marital status.

**Conclusion:**

TyG‐WHtR is a potential CMM predictor, and maintaining it within a moderate range may help prevent CMM.

## Graphical abstract

Using data from 6674 middle‐aged and older Chinese adults in the China Health and Retirement Longitudinal Study (CHARLS), this study investigated the association between the triglyceride glucose‐waist height ratio (TyG‐WHtR) index and cardiometabolic multimorbidity (CMM). Restricted cubic spline analysis revealed a nonlinear, U‐shaped association between TyG‐WHtR and CMM risk, with the lowest estimated risk observed at intermediate TyG‐WHtR levels. These findings suggest that maintaining TyG‐WHtR within a moderate range may be beneficial for CMM prevention and that TyG‐WHtR may serve as a practical marker for cardiometabolic risk assessment.

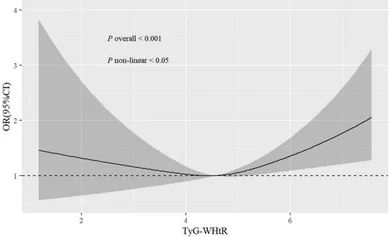

## 1 Introduction

Cardiometabolic diseases (CMDs) is the leading cause of premature human mortality worldwide, imposing an extremely high economic and health burden on society as a whole and on individuals. Cardiometabolic multimorbidity (CMM) is defined as the presence of at least two CMDs, usually including diabetes, heart disease, and stroke [1]. It has been discovered that having CMM is significantly linked to a higher risk of dying and a lower lifespan than having CMD alone [2, 3]. As China's population continues to age, the prevalence of CMM is steadily rising, with approximately 4.7% of the elderly suffering from CMM [4, 5, 6]. Previous research has focused on the exploration of certain diseases in CMM; therefore, early identification of people at high risk of CMM and timely implementation of early intervention and treatment are critical for preventing the occurrence of CMM and promoting overall well‐being.

TyG index is a novel index calculated from fasting triglyceride (TG) and fasting blood glucose concentration (FBG), which is closely related to insulin resistance (IR) [7]. TyG‐WHtR is a modified index based on TyG with the introduction of height and waist circumference, which has the advantages of simplicity, convenience, and ease of measurement, and it is a promising marker for the early identification of cardiovascular events and metabolic disorders [8, 9]. From a public health perspective, identifying simple, low‐cost, and readily accessible markers such as TyG‐WHtR may improve early risk stratification and targeted prevention in resource‐limited settings. TyG‐WHtR has been shown to have predictive value in previous studies, and Qiushi Ren et al. [10] found that changes in TyG‐WHtR were independently associated with the risk of cardiovascular disease (CVD). Most of the previous studies on CMM have focused on European and American populations [11, 12], and it is unclear whether the TyG‐WHtR index can be utilized as a reliable predictor of CMM. Evidence from Chinese populations remains limited, particularly among middle‐aged and older adults who are experiencing a rapidly increasing burden of cardiometabolic diseases. Therefore, using data from CHARLS, a nationally representative cohort of Chinese middle‐aged and older adults, this study may help address the geographical evidence gap and provide population‐specific evidence on the association between TyG‐WHtR and CMM. This study is a cohort study designed to evaluate the predictive value of TyG‐WHtR as a continuous variable. In addition, a stratified analysis revealed the influence of baseline parameters on these associations.

## 2 Methods

### 2.1 Study Population

Our data comes from CHARLS, a nationwide population‐based cohort study. CHARLS is a comprehensive national survey that provides a dependable database for studying the health of middle‐aged and older adults. This program received ethical approval from the Biomedical Ethics Review Board of Peking University.

In this cohort study, we analyzed baseline data from the 2011 wave in CHARLS (http://charls.pku.edu.cn/) and follow‐up data from CMM in the 2018 wave. In this study, we collected 11,847 participants from the CHARLS (2011) baseline data with the following exclusion criteria: (1) participants with less than 45 years old; (2) participants with missing socio‐demographic data in four categories: gender, education, marital status, and place of residence; (3) participants taking lipid‐lowering medications in 2011; (4) participants with insufficient information to ascertain CMM status at either the 2011 baseline survey or the 2018 follow‐up survey, including missing data on diabetes, heart disease, or stroke; and (5) participants with self‐reported confirmed CMM in the 2011 baseline data. Finally, we included 6674 participants.

### 2.2 Measurement

#### 2.2.1 Assessment of the Triglyceride Glucose‐Waist Height Ratio Index

Fasting blood glucose and triglyceride levels, waist circumference, and height data were obtained from 2011. TyG‐WHtR is a new type of indicator that combines human data (waist circumference, height) and laboratory indicators (blood glucose, triglycerides) and is calculated as follows:

1. TyG = ln [triglycerides (mg/dl) × glucose (mg/dl)/2].
2. WHtR = Waist circumference/height.
3. TyG‐WHtR = TyG × WHtR.

#### 2.2.2 Assessment of CMM

Stroke and heart disease were identified based on a self‐reported physician diagnosis. Diabetes mellitus (DM) was defined as follows [13]: fasting blood glucose (FBG) ≥ 126 mg/dL, and/or glycated hemoglobin (HbA1c) ≥ 6.5%, and/or a self‐reported physician diagnosis (a positive response to “Have you been diagnosed with DM?”), and/or recent use of hypoglycemic medication (insulin and/or any oral hypoglycemic drug). Heart disease was determined as a self‐reported physician diagnosis, indicated by a positive response to the question [14].

In this study, CMM was defined as the co‐occurrence of at least two of the following three cardiometabolic conditions: diabetes mellitus, heart disease, and stroke. Participants with CMM at baseline in 2011 were excluded. Therefore, incident CMM was ascertained at the 2018 follow‐up if participants had any two or all three of these conditions, regardless of the specific disease combination or the temporal sequence of disease onset.

#### 2.2.3 Assessment of Covariates

We analyzed age, gender, education level, location and marital status as demographic information. We analyzed smoking status as health‐related behavioral variables. We analyzed measured value variables including body mass index (BMI) and waist circumference (WC).

### 2.3 Statistical Analysis

Continuous variables are characterized using standard deviation (SD) or median [interquartile range (IQR)] ± mean, based on the data distribution. Categorical variables are presented as frequencies and percentages. Baseline characteristics and CMM incidence were compared using one‐way ANOVA, Kruskal‐Wallis H‐test, or chi‐square test. Three models have been constructed to investigate the link between TyG‐WHtR and CMM, and included unadjusted crude models (Model 1); adjusted models for age, sex, education, location, and marital status (Model 2); and further adjusted models for smoking status, BMI, and WC (Model 3). WC was included in Model 3 to examine whether the association between TyG‐WHtR and CMM was still present after further accounting for abdominal adiposity. As WC is part of the WHtR component of TyG‐WHtR, this model was considered a more conservative adjustment model. These results are expressed as OR and 95% CI. Restricted cubic spline tests for possible nonlinear associations were used and dose–response associations for TyG‐WHtR and CMM were visualized. All statistical analyses were done with r 4.1. Restricted triplicates were completed using the ‘rms’ package.

## 3 Results

### 3.1 Study Sample

The baseline characteristics of participants are shown in Table 1. The overall number of participants was 6674 (median age 58.1 years, 3043 (45.6%) males, 3631 (54.4%) females, of whom 1140 (17.08%) developed CMM). The baseline median (IQR) TyG‐WHtR for all participants was 4.4 (1.0). The difference between those with and without CMM was significant, with older age, female, unmarried, and high waist circumference being more likely to develop CMM.

**TABLE 1:** Characteristics of the participants at baseline (*N* = 6674).

Stratified by CMM				
	Total, (*n* = 6674)	Non‐CMM (*n* = 5534)	CMM, (*n* = 1140)	*p*
TyG‐WHtR (mean [SD])	4.6 (1.1)	4.5 (0.9)	5.0 (1.7)	< 0.001
Age (mean [SD])	58.1 (8.8)	57.7 (8.8)	60.1 (8.6)	< 0.001
Gender (%)				
Female	3631 (54.4)	2958 (53.5)	673 (59.0)	0.001
Male	3043 (45.6)	2576 (46.5)	467 (41.0)	
Marital (%)				
Married	5968 (89.4)	4981 (90.0)	987 (86.6)	0.001
Single	706 (10.6)	553 (10.0)	153 (13.4)	
Smoking (%)				
Current smoker	2072 (31.1)	1778 (32.2)	294 (25.8)	< 0.001
Ex‐smoker	491 (7.4)	383 (6.9)	108 (9.5)	
Non‐smoker	4100 (61.5)	3364 (60.9)	736 (64.7)	
Education (%)				
High school and above	648 (9.7)	538 (9.7)	110 (9.6)	< 0.001
Illiterate	1935 (29.0)	1546 (27.9)	389 (34.1)	
Middle school	1351 (20.2)	1156 (20.9)	195 (17.1)	
Primary school	2740 (41.1)	2294 (41.5)	446 (39.1)	
Location (%)				
Rural	4510 (67.6)	3758 (67.9)	752 (66.0)	0.215
Urban	2164 (32.4)	1776 (32.1)	388 (34.0)	
BMI (%)				
Normal	4231 (64.2)	3664 (67.0)	567 (50.7)	< 0.001
Obesity	276 (4.2)	185 (3.4)	91 (8.1)	
Overweight	1632 (24.8)	1217 (22.2)	415 (37.1)	
Underweight	450 (6.8)	404 (7.4)	46 (4.1)	
WC (mean (SD))	83.7 (12.1)	82.9 (11.7)	87.7 (13.4)	< 0.001
Quartiles of TyG‐WHtR (%)				
Q1	1669 (25.0)	1516 (27.4)	153 (13.4)	< 0.001
Q2	1668 (25.0)	1455 (26.3)	213 (18.7)	
Q3	1668 (25.0)	1394 (25.2)	274 (24.0)	
Q4	1669 (25.0)	1169 (21.1)	500 (43.9)	
Quartiles of TyG‐WHtR (%)	4.4 (1.0)	4.4 (0.9)	4.8 (1.6)	< 0.001

Abbreviations: BMI, body mass index; TyG‐WHtR, triglyceride glucose‐waist height ratio index; WC, waist circumference.

### 3.2 The Relationship Between TyG‐WHtR and Cardiometabolic Multi‐Morbidity

Table 2 demonstrates the relationship between TyG‐WHtR and CMM risk. The risk of CMM rose with TyG‐WHtR quartiles (p for trend < 0.001). In Model 2, participants in the fourth TyG‐WHtR quartile group (Q4) had the highest risk of CMM after adjusting demographic information (OR, 1.23, 95% CI, 1.20–1.26).

**TABLE 2:** Association of TyG‐WHtR with the risk of CMM in the CHARLS.

	Model 1	*p*	Model 2	*p*	Model 3	*p*
TyG‐WHtR per IQR	1.06 (1.05, 1.07)	< 0.001	1.06 (1.05, 1.07)	< 0.001	1.02 (1.01, 1.04)	< 0.001
Quartiles of TyG‐WHtR						
Q1	Ref		Ref		Ref	
Q2	1.04 (1.01, 1.06)	0.005	1.04 (1.01, 1.06)	0.004	1.03 (1.00, 1.06)	0.052
Q3	1.08 (1.05, 1.10)	< 0.001	1.08 (1.05, 1.10)	< 0.001	1.04 (1.01, 1.08)	0.013
Q4	1.23 (1.20, 1.26)	< 0.001	1.23 (1.20, 1.26)	< 0.001	1.15 (1.11, 1.20)	< 0.001
*p* for trend	< 0.001		< 0.001		< 0.001	

*Note:* Model 1 was crude model. Model 2 was adjusted for age, gender, education level, family residence, and marital. Model 3 was adjusted for age, gender, education level, family residence, marital, smoking, BMI and WC.

Abbreviations: BMI, body mass index; CHARLS, China Health and Retirement Longitudinal Study; CMM, cardiometabolic multimorbidity; IQR, interquartile range; TyG‐WHtR, triglyceride glucose‐waist height ratio index; WC, waist circumference.

Figure 1 demonstrates the association between TyG‐WHtR and CMM4 and its nonlinear trend. Restricted cubic spline regression showed a significant overall effect of the association between TyG‐WHtR and CMM risk (p overall < 0.001) and a non‐linear (U‐shaped) relationship between the two (p non‐linear < 0.05).

**FIGURE 1:**
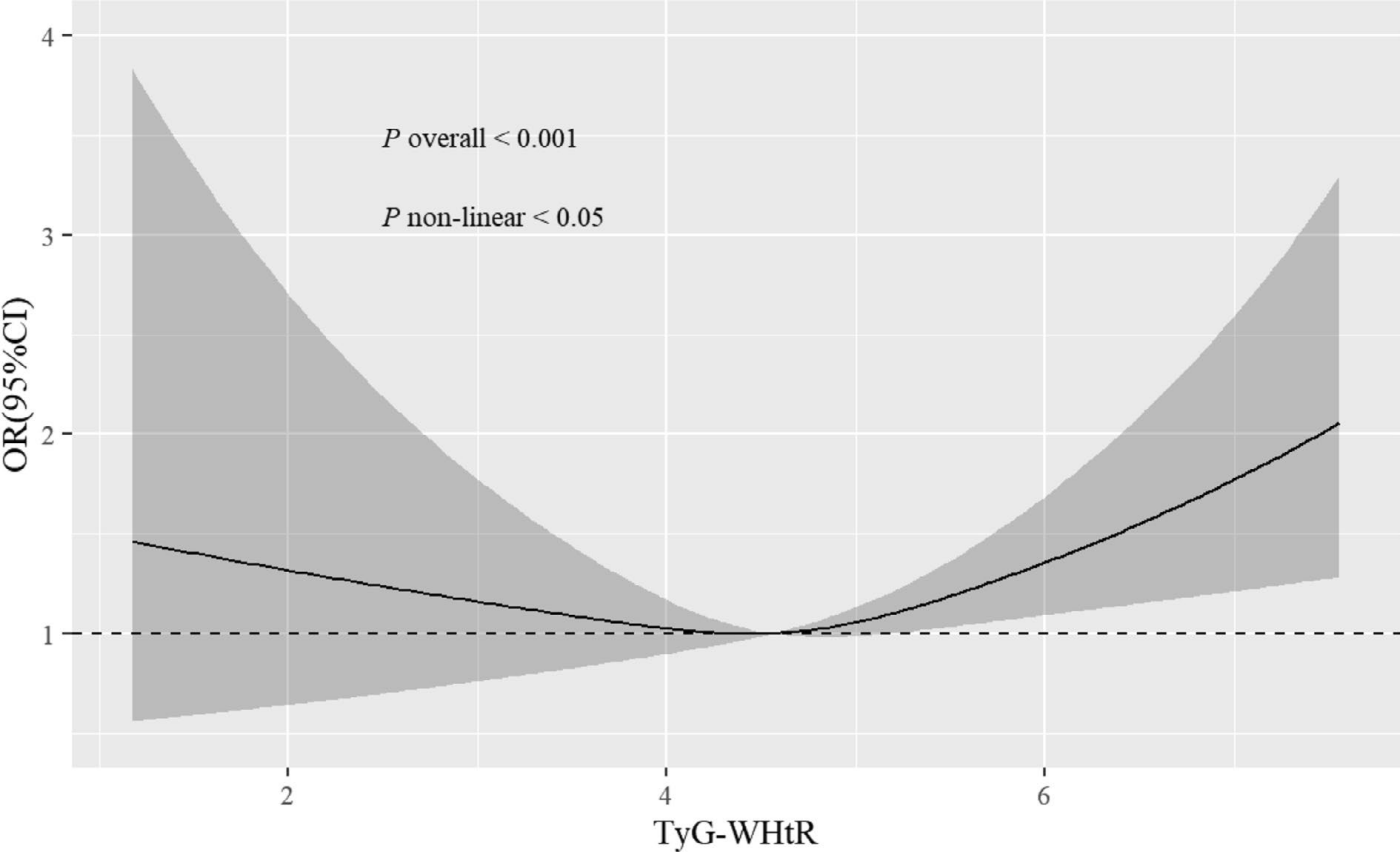
Restricted cubic splines were used to test the hypothesis of nonlinear associations between the TyG‐WHtR index and CMM. The model was adjusted for age, gender, education level, location, marital status, smoking status, BMI, and WC. The plot shows a non‐linear (U‐shaped) relationship between the TyG‐WHtR index and the risk of CMM. TyG‐WHtR, triglyceride glucose‐waist height ratio index; CMM, cardiometabolic multi‐morbidity; WC, waist circumference. BMI, body mass index.

### 3.3 Stratified Analysis

To determine whether the association between TyG‐WHtR and CMM differed across subgroups, participants were stratified by baseline characteristics. As shown in Figure 2, higher TyG‐WHtR was positively associated with CMM risk in several subgroups, including females and ex‐smokers. As presented in Table 3, stratified analyses showed significant interactions between TyG‐WHtR and age (*p* = 0.03), marital status (*p* = 0.02), place of residence (*p* < 0.001), and waist circumference (*p* < 0.001), suggesting that the association between TyG‐WHtR and CMM may vary across these subgroups.

**FIGURE 2:**
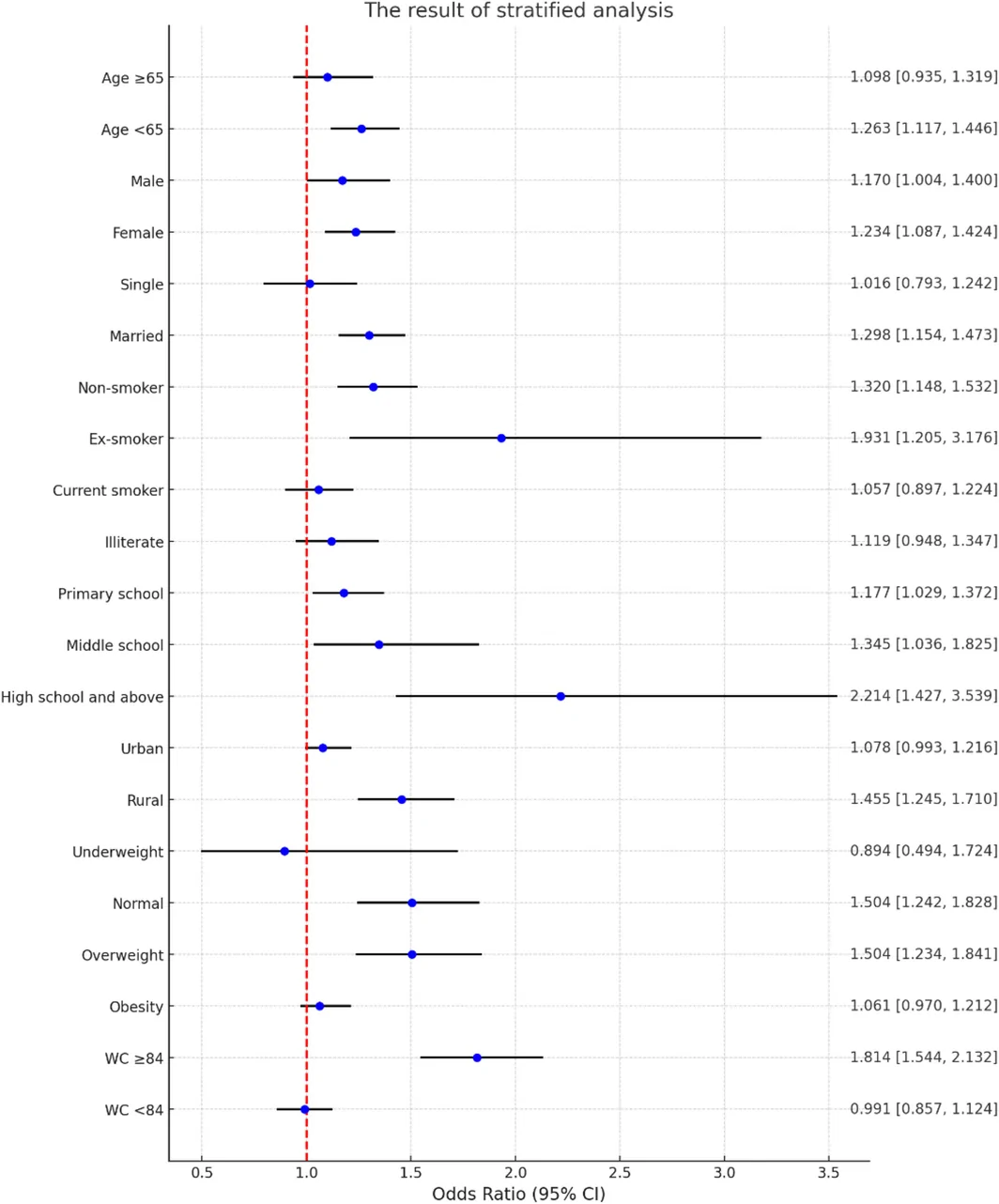
Forest plot of stratified analysis of the association of TyG‐WHtR index with the risk of CMM. OR, odds ratio; CI, confidence intervals; TyG‐WHtR, triglyceride glucose‐waist height ratio index; CMM, cardiometabolic multi‐morbidity.

**TABLE 3:** Analysis of interaction effects.

Covariates	*p* for interaction
Age	0.03
Gender	0.28
Marital	0.02
Education	0.20
Location	< 0.001
Smoking	0.18
BMI	0.93
WC	< 0.001

## 4 Discussion

In this cohort study based on 6674 participants, we discovered a substantial correlation between the TyG‐WHtR index and the risk of CMM. Each one‐unit increase in IQR of TyG‐WHtR was linked to a 23% rise in the chance of getting CMM (OR, 1.23, 95% CI, 1.20–1.26). We found a nonlinear (U‐shaped) relationship between the TyG‐WHtR index and the risk of CMM. The TyG‐WHtR index is a potential predictor of CMM, and controlling it within a moderate range may help to prevent CMM.

TyG‐WHtR is a novel index that incorporates height and waist circumference into the calculation based on the TyG index. TyG index is closely related to IR [15, 16]. CMD development is significantly influenced by imbalances in lipid and glucose metabolism, with IR as a core component [17, 18]. Studies have shown an association between body fat distribution and CMD trajectory [19]. Other research has discovered that obesity is pivotal in causing and intensifying CVD. Overweight and obesity are not only important determinants of CVD, but also primary causes of the type 2 diabetes (T2DM) epidemic [20, 21]. WHtR is a more significant indicator of body fat distribution as well as obesity compared to WC, and could be a more effective substitute for the TyG index. Yuchen Tang et al. revealed that TyG‐WHtR had significant accuracy in identifying arterial stiffness in Chinese who are overweight or obese [22].

IR is an important etiological factor in T2DM, which manifests itself as a failure of glucose to enter cells efficiently due to a weakened cellular response to insulin. IR also exacerbates the onset and progression of atherosclerosis and independently correlates with an elevated risk of cardiovascular disease development [23]. The metabolic score for insulin resistance may be a useful predictive indicator of mortality risk in patients with CVD, according to a cohort research [24]. TyG is a reliable biomarker of IR with excellent sensitivity and specificity [25]. Anxiang Li et al. found that WtHR (Waist circumference/height) had the ability to predict IR and could be a preferred indicator for screening IR based on a cross‐sectional study conducted in three Chinese hospitals [26]. Qi Feng et al. found that WHtR has a linear relationship with ischaemic cardiovascular disease, heart attacks, and ischaemic strokes when used as a proxy for centripetal obesity [27].

The results of subgroup analyses showed gender disparity. Females are more vulnerable to CMM than males. Estrogen regulates insulin sensitivity and serves an essential function in the maintenance of metabolic homeostasis [28]. Estrogen levels decline rapidly after menopause in women. According to reports, men are more likely than women to have a stroke, but this prevalence reverses after menopause, with more women than men suffering from stroke [29, 30]. Atherosclerosis accelerates rapidly in postmenopausal women, which is accompanied by an increased prevalence of coronary artery disease [31]. The positive association observed among ex‐smokers in the stratified analysis deserves further consideration. However, the interaction between TyG‐WHtR and smoking status was not statistically significant (P for interaction = 0.18), suggesting that the association did not statistically differ across smoking subgroups. This finding may partly reflect the “sick quitter” phenomenon, in which individuals with metabolic abnormalities or early cardiometabolic symptoms are more likely to quit smoking after becoming aware of their health status or receiving medical advice [32]. Thus, ex‐smokers may have a higher underlying metabolic burden than never‐smokers or current smokers. In addition, previous smoking exposure may exert adverse metabolic effects, while post‐cessation weight gain or transient changes in glucose metabolism may further strengthen the association between TyG‐WHtR and CMM [33, 34]. Given the lack of detailed information on smoking intensity, time since cessation, reasons for quitting, and post‐cessation weight change, this subgroup finding should be interpreted cautiously.

The significant interactions observed for location and marital status also deserve attention. The stronger association between TyG‐WHtR and CMM in rural participants may be partly explained by differences in socioeconomic status, lifestyle patterns, health literacy, and access to medical services [35, 36]. Rural residents may have fewer opportunities for early screening and management of metabolic abnormalities, which could increase the likelihood that elevated TyG‐WHtR translates into subsequent CMM. Marital status may also modify this association through shared household dietary habits, lifestyle routines, and social support, as marital status has been associated with cardiovascular risk in previous studies [37]. However, these subgroup findings should be interpreted as exploratory, and further studies incorporating socioeconomic, behavioral, and healthcare‐related factors are needed to clarify the underlying mechanisms.

According to our results, there is a U‐shaped connection between the TyG‐WHtR index and CMM risk, which means that controlling the TyG‐WHtR index within a reasonable range can contribute to decreasing the risk of CMM, and too low or too high a value of TyG‐WHtR can increase the risk of developing CMM.

As a prospective cohort study based on the national population, this research encompasses a broad spectrum of participants with excellent sample representation. By focusing on Chinese middle‐aged and older adults, our study extends previous evidence largely derived from Western populations and provides additional data for understanding CMM risk prediction in a Chinese population. Despite many advantages, the study faces some potential limitations. To begin with, the participants in our study were solely from the older Chinese population, suggesting that these results might not be applicable to different ethnicities or age ranges. Second, heart disease and stroke were identified based on self‐reported physician diagnoses, which may have introduced recall bias. Third, despite extensive adjustments for variables, there is still a chance of residual confusion. Fourth, the interpretation of Model 3 requires some caution. WC was included in this model to further account for the potential influence of abdominal adiposity. However, given the mathematical overlap between WC and the WHtR component of TyG‐WHtR, potential collinearity or overadjustment cannot be completely excluded. Therefore, the WC‐adjusted estimates should be regarded as conservative, and the attenuation of the ORs in Model 3 should be interpreted in this context. Finally, as a cohort study, we were only able to obtain correlational findings and were unable to establish a causal relationship between the TyG‐WHtR index and CMM.

## 5 Conclusion

In summary, a higher TyG‐WHtR index was closely associated with the occurrence of CMM among Chinese middle‐aged and older adults, with a nonlinear dose–response relationship. These findings suggest that maintaining TyG‐WHtR within an appropriate range and monitoring it at an early stage may help identify individuals at higher cardiometabolic risk and support timely preventive interventions. Because TyG‐WHtR can be calculated from routine anthropometric and laboratory measurements, it may serve as a simple and practical marker for cardiometabolic risk screening in primary care and community‐based settings. Compared with the TyG index or WHtR alone, TyG‐WHtR combines information on glucose‐lipid metabolism and central adiposity, which may help capture cardiometabolic risk more comprehensively. Further studies are needed to directly compare its predictive performance with other established indices for CMM.

## Author Contributions

**Lingling Li:** conceptualization, software, writing – original draft, writing – review and editing. **Zongliang Yu:** conceptualization, data curation, writing – original draft, writing – review and editing. **Yixuan Feng:** conceptualization, project administration, writing – original draft, writing – review and editing. **Changxin Sun:** conceptualization, investigation, writing – review and editing. **Lanqing Hu:** conceptualization, validation, writing – review and editing. **Xinyi Liu:** conceptualization, validation, writing – review and editing. **Liang Li:** resources, writing – review and editing. **Min Wu:** resources, supervision, writing – review and editing. **Longtao Liu:** funding acquisition, supervision, writing – review and editing.

## Funding

This study was supported by National Natural Science Foundation of China (No. 82575045, China), and National Natural Science Foundation of China (No. 82374281, China), and Scientific and Technological Innovation Project of China Academy of Chinese Medical Sciences (No. CI2026A00910, China), and High Level Chinese Medical Hospital Promotion Project (No. HLCMHPP20230150, China).

## Ethics Statement

This program received ethical approval from the Biomedical Ethics Review Board of Peking University, under approval numbers IRB00001052‐11015 and IRB00001052‐11014. Every participant gave written informed permission.

## Conflicts of Interest

The authors declare no conflicts of interest.

## Data Availability

The data that support the findings of this study are available in China Health and Retirement Longitudinal Study (CHARLS) at http://charls.pku.edu.cn/. These data were derived from the following resources available in the public domain: National Baseline Survey in 2011, https://charls.charlsdata.com/pages/Data/2011‐charls‐wave1/zh‐cn.html; National Baseline Survey in 2018, https://charls.charlsdata.com/pages/Data/2018‐charls‐wave4/zh‐cn.html.
